# Effect of anaemia on hand grip strength, walking speed, functionality and 1 year mortality in older hospitalized patients

**DOI:** 10.1186/s12877-016-0326-y

**Published:** 2016-08-19

**Authors:** Etienne Joosten, Elke Detroyer, Koen Milisen

**Affiliations:** 1 Department of Internal Medicine, Division of Geriatric Medicine, University Hospitals Leuven, Leuven, Belgium; 2 Department of Public Health and Primary Care, Academic Centre for Nursing and Midwifery, KU Leuven, Leuven, Belgium

**Keywords:** Anaemia, Hand grip strength, Gait speed, Activities of daily living, Hospitalized older patients

## Abstract

**Background:**

Anaemia is a common problem in hospitalized older patients and is recognized as a risk factor for a significant number of adverse outcomes. Data of the effect of anaemia on functional status during hospitalization and mortality after discharge are limited. Aim of the study is to examine whether there is an association between anaemia, hand grip strength, gait speed and basic activities of daily living (ADL) during hospitalization and mortality 1 year after discharge in geriatric patients.

**Methods:**

In a prospective study, data on age, sex, body mass index, Mini-Mental State Examination (MMSE), main clinical diagnosis, number of comorbidities, hand grip strength, gait speed, ADL, haemoglobin, C-reactive protein and estimated Glomerular filtration ratio (eGFR) were recorded in 220 older patients, admitted to the acute geriatric ward of a university hospital. Anaemia was defined as a haemoglobin level <13 g/dL for men and <12 g/dL for women and was further specified into severe (haemoglobin level <10 g/dL for both men and women) and moderate anaemia (haemoglobin between 10 and 12 g/dL for women and 10 and 13 g/dL for men). Gait speed (in meters per second) was calculated after a 4.5 m walk and hand grip strength (in kilogram) was assessed with a hydraulic hand dynamometer. Functionality was assessed in the six basic activities of daily living. Information about the vital status was obtained 1 year after discharge with a telephone call. Analysis of covariance (ANCOVA) was used to examine the effect of the anaemia status on the walking speed, hand grip strength and premorbid ADL index and logistic regression analysis was used to examine whether anaemia could be identified as risk factors for mortality 12 months after discharge.

**Results:**

Overall, 106 (48 %) patients had anaemia. Hand-grip strength, gait speed and ADL score were not significantly different between anaemic and non-anaemic hospitalized geriatric patients. After adjustment for age, sex, body mass index, eGFR, MMSE, number of comorbidities and main clinical diagnosis, the means for hand-grip strength were 17.3, 19.9 and 19.1 kg (*p* = 0.38); for gait speed 0.57, 0.52 and 0.47 m/s (*p* = 0.28); and for the ADL score 3.50, 3.05 and 3.30 (*p* = 0.75) in patients with severe, moderate and without anaemia, respectively. In the unadjusted model, the odds ratio for mortality 1 year after discharge was 2.72 (95 % CI 1.20–6.14) and 4.70 (95 % CI 1.91–11.77) for moderate and severe anaemia, respectively, with no anaemia as the reference group. After adjustment for several confounders, a haemoglobin level less than 10 g/dl (OR 3.87; 95 % CI 1.25–11.99) remained significantly associated with an increased mortality over that 1 year period.

**Conclusion:**

Our results do not support that anaemia on admission is associated with a decline in physical performance (hand grip strength and gait speed) and functionality (ADL) during hospitalization in older patients. However, severe anaemia is a significant risk factor for an increased mortality over a 1 year period after discharge.

## Background

Anaemia is a frequent clinical problem in older patients and is commonly defined according to the WHO criteria as a haemoglobin value less than 12 g/dL in women and less than 13 g/dL in men [1]. The prevalence of anaemia varies widely according to the diagnostic criteria and the patient population. As a consequence, the anaemia prevalence defined according to the WHO criteria ranges from 8.1 to 24.7 % in community-dwelling older persons, from 31 to 60 % in nursing home patients and from 40 to 72 % in hospitalized older patients [2–6]. Anaemia is recognized as a risk factor for a number of adverse outcomes in older people such as increased risk for hospitalization and mortality, falls, cognitive impairment, decreased physical performance and muscle strength and poorer recovery from activity of daily living disability but the exact underlying mechanism that might explain these associations is unclear [4, 7–11]. Most of these studies were performed in community-living older persons and studies reporting on the association between anaemia, gait speed, muscle strength and functionality in hospitalized older patients are rare. The aim of the present study was to examine the relationship between anaemia and gait speed, hand grip strength and disability in basic activities of daily living in hospitalized older patients. We also investigated whether anaemia was associated with an increased mortality risk during a 12 month period after discharge.

## Methods

This study is part of a broader prospective study on frailty and delirium in hospitalized older patients. Details of the study design, recruitment, exclusion criteria, geriatric assessments and patient characteristics were described elsewhere [12]. Briefly, 511 consecutive older patients aged 70 years and older and admitted to the acute geriatric ward of a university hospital were invited to participate of which 291 patients were excluded for various reasons [12]. Hence, the final sample comprised 220 patients. For this study, physical function and functional status were the primary geriatric assessments. Measures of physical function included gait speed (in meters per second) and hand grip strength (in kg) and were assessed 24 h after admission as part of the Cardiovascular Health Study criteria [13]. A gait speed less than 0.8 m/s for men and women was defined as low and a grip strength less than 26 kg for men and less than 16 kg for women was classified as weak [14]. The functional [15] and the cognitive status [16], analysis of the blood samples (haemoglobin, C-reactive protein, serum creatinine), calculation of the estimated glomerular filtration rate (eGFR) and body mass index (kg/m2) were performed as described previously [12]. Functional decline was defined as an increase of at least 1 point on the ADL index between the premorbid evaluation on admission (performed during the last 2 weeks before hospitalization) and at discharge. Anaemia was defined according to the WHO criteria (haemoglobin <13 g/dL for men and <12 g/dL for women) and further classified into severe (haemoglobin level <10 g/dL for both men and women) and moderate anaemia (haemoglobin between 10 and 12 g/dL for women and between 10 and 13 g/dL for men). The number of comorbidities was calculated as previously described [12, 17]. Information on the vital status (deceased or alive) 12 months after discharge was obtained by phone by one of the research nurses and came from the patient, a family member or a proxy. The number of deaths was defined as the patients who died over a 12 month period after discharge.

The Medical Ethics Committee of the Leuven University Hospitals approved the study and informed (proxy) consent was obtained for each participant before inclusion.

### Statistical methods

The Kolmogorov-Smirnoff test was used to investigate the normal distribution of the data. Comparison between two groups was carried out by the student’s t test or the Mann-Whitney U test, depending on the parametric or non-parametric distribution of the data. Categorical variables were compared with the Chi-square test and the Fisher Exact test. A Spearman’s rank-order correlation was run to determine the relationship between haemoglobin, hand grip strength and walking speed. Analysis of covariance (ANCOVA) was used to examine the effect of the anaemia status (severe, moderate, no anaemia) on the walking speed, hand grip strength and premorbid ADL index adjusted for sex, age, eGFR, BMI, MMSE, clinical diagnosis and number of comorbidities. Logistic regression analysis was used to examine whether moderate and severe anaemia as compared to no anaemia could be identified as risk factors for mortality 12 months after discharge, adjusted for the specific covariates age, sex, BMI, MMSE, eGFR, functional decline and number of comorbidities. All statistical analyses were performed with SPSS, version 22 (SPSS Inc.). *P* values <0.05 were considered to be statistically significant.

## Results

The baseline results of the study population are shown in Table 1. The mean age of the 220 patients was 83.6 years (±5.0) and 57 % were female. Table 1 shows the main clinical diagnoses on admission in relationship to the anaemia status. The mean number of comorbidities per patient was 2.62 (±1.7) (median number two with a range of 0–12) and the most prevalent diseases were congestive heart failure, chronic kidney diseases, chronic pulmonary diseases, diabetes mellitus, collagenous diseases including osteoarthritis and dementia. A total of 106 (48 %) patients were anaemic of which 37 (17 %) had severe and 69 (31 %) moderate anaemia. Patients with anaemia were more likely to be male, had a higher burden of comorbidities (*p* = 0.001) and a significantly lower eGFR (*p* <0.001) (Table 1). The premorbid ADL scores and ADL scores at discharge were not statistically different between anaemic and non-anaemic patients (Table 1). An accurate measurement of the hand grip strength was available in 209 patients, 91 males and 118 females. A weak handgrip strength (<26 kg for men and <16 kg for women) was found in 54 (59 %) men and 65 (55 %) women. The mean handgrip strength was significantly higher in men (mean 23.1 ± 9.8 kg) then women (mean 14.7 ± 7.4 kg) (*p* <0.001) but was comparable between anaemic (mean 18.4 ± 9.8 kg) and non-anaemic (mean 18.3 ± 9.2 kg) patients (*p* = 0.75) (Table 1). Although 200 patients started the 4.5 m gait speed walk, only 97 (47 men, 50 women) completed this test. Only nine of these 97 patients had a gait speed higher than 0.8 m/s. The mean gait speed was comparable between men (mean 0.51 ± 0.25 m/s) and women (0.47 ± 0.2 m/s) (*P* = 0.55) and between anaemic (0.51 ± 0.22 m/s) and non-anaemic (mean 0.47 ± 0.23 m/s) patients (*p* = 0.36) (Table 1).

**Table 1:** Patient characteristics according to the anaemia status

	Anaemia (*n* = 106)	Non-anaemia (*n* = 114)	*P*
Sex (M/F)	56/50	38/76	0.003
Age (y), mean (SD)	84.04 (5.1)	83.1 (4.9)	0.21
BMI (kg/m^2^), mean (SD)	25.5 (5.0)	25.9 (5.4)	0.71
ADL, premorbid at admission, mean (SD)	3.74 (3.18)	3.06 (3.17)	0.09
^a^ADL, at discharge, mean (SD)	3.87 (3.28)	3.51 (3.10)	0.49
CRP (mg/L), mean (SD)	49.3 (73.2)	39.5 (67.8)	0.15
eGFR (ml/min/1.73 m^2^), mean (SD)	46.6 (23.1)	58.5 (21.9)	<0.001
MMSE, mean (SD)	8.2 (3.4)	8.5 (2.8)	0.77
Main clinical diagnosis, n			
-Heart and respiratory failure	8	16	0.135
-Infectious diseases	31	27	0.35
-Cancer	9	1	0.008
-Gastrointestinal diseases	17	14	0.424
-Falls-fractures-osteoporosis	10	27	0.005
-Neuropsychiatric diseases	8	11	0.58
-Other	23	18	0.26
LOS (days), mean (SD)	16.4 (13.8)	15.7 (11.2)	0.88
Comorbidities, mean (SD)	3.13 (1.9)	2.3 (1.5)	0.001
^b^Hand grip strength (kg), mean (SD)	18.4 (9.8)	18.3 (9.2)	0.75
^c^Gait speed (m/sec), mean (SD)	0.51 (0.22)	0.47 (0.23)	0.36

*Abbreviations*: *M* male, *F* female, *BMI* body mass index, *ADL* activities of daily living (score between 0 and 12, higher scores indicating greater dependency), *CRP* C-reactive protein, *eGFR* estimated glomerular filtration ratio, *MMSE* Mini-Mental State Examination (maximum score of 12 as the best result), *LOS* length of stay

^a^Data for ADL at discharge were available in 191 patients (83 patients with and 108 without anaemia)

^b^Data for hand grip strength were available in 209 patients (104 patients with and 105 without anaemia)

^c^Data for gait speed were available in 97 patients (42 patients with and 55 patients without anaemia)

No significant correlation was found between the haemoglobin values and the hand-grip strength (Spearman’s rho 0.112, *p* = 0.1) and walking speed (Spearman’s rho 0.04, *p* = 0.69).

In order to determine whether anaemia was related to the premorbid ADL score, handgrip strength and gait speed, ANCOVA was run after adjustment for sex, age, MMSE, eGFR, clinical diagnosis and number of comorbidities. The adjusted means for the ADL score, hand grip strength and walking speed were not significantly different between the subgroups with severe anaemia, moderate anaemia and no anaemia (Table 2). Ten of the 220 patients died during the index hospitalization. The ADL scores at discharge were available in 191 of the 210 remaining patients and a functional decline during hospitalisation was demonstrated in 77 (40.3 %) of these patients: 46 of the 108 non-anaemic and 31 of the 83 anaemic patients (*p* = 0.46). Information on the vital status 1 year after discharge was available in 200 out of the 210 patients and 42 of them were deceased. In order to investigate whether anaemia is a risk factor for mortality 1 year after discharge, a logistic regression model was used to evaluate the association between mortality as the dependent variable and anaemia (severe, moderate, no anaemia) as the independent variable (Table 3). We controlled for other confounding variables: sex, age, BMI, MMSE, eGFR, functional decline and the number of comorbidities. In the unadjusted model, the odds ratio for mortality 1 year after discharge for patients with severe and moderate anaemia during the index hospitalization was 4.7 (95 % CI 1.91–11.77) and 2.72 (95 % CI 1.2–6.14), respectively, as compared to participants without anaemia (Table 3). After adjustment for the different covariates, only severe anaemia remained a significant risk factor for mortality 1 year after discharge with an odds ratio of 3.87 (95 % CI 1.25–11.99) (Table 3).

**Table 2:** Adjusted mean handgrip strength, walking velocity and ADL according to the severity of the anaemia

	Severe anaemia (*n* = 37)	Moderate anaemia (*n* = 69)	No anaemia (*n* = 114)	*P*
Adjusted mean premorbid ADL, score	3.50 (2.5–4.47)	3.05 (2.30–3.80)	3.30 (2.71–3.88)	0.75
^a^adjusted mean handgrip strength, kg	17.3 (14.4–20.2)	19.9 (17.7–22.1)	19.1 (17.3–20.8)	0.38
^b^adjusted mean gait speed, m/s	0.57 (0.45–0.70)	0.52 (0.43–0.60)	0.47 (0.4–0.54)	0.28

Data presented as mean (95 % CI) and adjusted for sex, age, body mass index, mini mental state examination, estimated glomerular filtration ratio, clinical diagnosis and number of comorbidities

*Abbreviations*: *ADL* activities of daily living

Severe anaemia: haemoglobin <10 g/dL; moderate anaemia: haemoglobin between 10 and 12 g/dL for women and between 10 and 13 g/dL for men; no anaemia: haemoglobin ≥12 g/dL for women and ≥13 g/dL for men

^a^Data for hand grip strength were available in 209 patients (104 patients with and 105 without anaemia)

^b^Data for gait speed were available in 97 patients (42 patients with and 55 patients without anaemia)

**Table 3:** Anaemia and the risk of mortality 1 year after hospitalization^a^

	Unadjusted OR	Adjusted OR ^b^
No anaemia	1	1
Moderate anaemia	2.72 (1.20–6.14); *p* = 0.016	2.04 (0.76–5.49); *p* = 0.16
Severe anaemia	4.70 (1.91–11.77); *p* = 0.001	3.87 (1.25–11.99); *p* = 0.02

*Abbreviations*: *OR* odds ratio (95 % confidence interval)

Severe anaemia: haemoglobin value <10 g/dL; moderate anaemia: haemoglobin between 10 and 12 g/dL for women and between 10 and 13 g/dL for men; no anaemia: haemoglobin ≥12 g/dL for women and ≥13 g/dL for men

^a^data were available in 191 patients

^b^adjusted for age, sex, body mass index, mini mental state examination, estimated glomerular filtration rate, number of comorbidities and functional decline (see methods)

## Discussion

We found that the hand-grip strength, gait speed, premorbid ADL score and ADL score at discharge were not significantly different between anaemic and non-anaemic hospitalized geriatric patients. After adjustment for age, sex, BMI, MMSE, eGFR, number of comorbidities and clinical diagnosis, the means for the ADL score, hand-grip strength and gait speed were similar between patients divided into those with severe, moderate and without anaemia. In addition, our data add some evidence to the importance of severe anaemia as a significant risk factor for the 1 year mortality after discharge. Anaemia is a common condition in hospitalized geriatric patients and its prevalence varies widely depending on the population studied and the different diagnostic criteria [1–6]. In this study, 48 % had anaemia according to the WHO criteria and this is in accordance with other studies [2, 4, 18]. Anaemia has been demonstrated to be significantly associated with a number of negative outcomes such as an increased mortality and hospitalization, poorer physical and cognitive performance, diminished quality of life, increased frailty and number of falls [7–9, 19–23]. Most studies were performed in community-dwelling older patients and the specific relationship between anaemia and muscle strength and gait speed have been investigated in a few studies. Penninx et al. and Haslam et al. found that anaemia was associated with a lower hand-grip strength and leg strength [7, 11]. In another study, self-reported mobility difficulty prevalence was lowest at haemoglobin concentrations around 14 g/dl and increased as the haemoglobin levels decreased toward 8 g/dl in older community-dwelling women [24]. An association between anaemia and functional disability, mostly basic and instrumental activities of daily living, has been shown in some studies [10, 25–28] but was less clear or absent in other studies [11, 23, 29, 30] and Maraldi et al. demonstrated that haemoglobin levels were positively associated with the likelihood of recovery from ADL disability in hospitalized older persons [4]. Most of the data are epidemiologic in nature and do not prove a causal relationship between anaemia and the different poor health outcomes. Although the underlying mechanisms that might explain the possible association between anaemia and physical decline are unclear, some hypotheses dealing with a diminished oxygen delivery to specific organs that jeopardize their functional capacity (i.e. the cardiorespiratory, musculoskeletal and neurological system) or anaemia as an of important marker of inflammation, have been put forward [31, 32]. However, our data do not support an association between anaemia and physical performance (hand grip strength and gait speed) and functionality (ADL). A possible explanation might be the patient selection in different studies. The mean age of our study population is higher than in most other studies and most patients admitted to the acute geriatric ward are by its nature frail or prefrail which increases the functional dependency as demonstrated by the fact that only a very small minority had a gait speed higher than 0.8 m/s. Some specific and commonly accepted anaemia-related symptoms and diseases such as fatigue and weakness but also chronic kidney disease, cancer, stroke, infectious and inflammatory disorders are also common in non-anaemic hospitalized older patients and could contribute to some extent to a diminished functionality and a more sedentary lifestyle. Although anaemic patients had more comorbidities in our study, the mean CRP as a marker of inflammation was comparable between the anaemic and non-anaemic group and the prevalence of falls and fractures was even higher in the latter group. All this might explain, at least partially, why we could not demonstrate that anaemia upon admission was a significant risk factor for functional disability, lower gait speed and muscle strength in hospitalized older patients because the majority of the patients were too sick, whether they were anaemic or not. Another explanatory factor could be that the functional assessments vary in a more dynamic way in hospitalized patients due to acute illnesses and their recovery as compared to non-hospitalized subjects. The mean gait speed and handgrip strength in our population were much lower as compared to non-hospitalized older patients but our results correspond well with other studies investigating hospitalized older persons [33, 34]. Unfortunately, data about the functional recovery after the index hospitalization in our patients are lacking. Our data also demonstrate that severe anaemia is a strong independent risk factor associated with an increased 1 year mortality after discharge and this is in accordance with the results of prior studies in community-dwelling older persons [9, 19, 20].

We are aware that our study has several limitations. We investigated a selected group of hospitalized older patients and chose some major and frequently used confounding variables, excluding other potential explanations linking anaemia with functionality. As a consequence, our results cannot be generalized to other patient groups. The extensive assessment of functionality in geriatric hospitalized patients is a time-consuming and complex procedure. Moreover, the handgrip strength and gait speed are both focused on the musculoskeletal system and require a minimum of the patients’ cooperation and physical skills which are often lacking in this vulnerable population. This limits the participation rate and as a consequence, the appropriate interpretation of gait speed was available in only 44 %. We are aware that the number of participants is limited but it is inherent to this population that the cooperation is also limited and the dropout high. Finally, it was not our intention in this study to diagnose the specific underlying cause for the anaemia (acute or chronic, anaemia of inflammation, iron deficiency anaemia etc.) but it is possible that the results of the geriatric assessment and the prognostic significance of anaemia differ according to the underlying anaemia aetiology.

## Conclusion

Our results do not support that anaemia on admission is associated with a decline in physical performance (hand grip strength and gait speed) and poorer functionality (ADL score) in hospitalized older patients. The reasons for these findings remain unclear but patient selection and a generalized vulnerability inherent for the population under study could contribute to a possible explanation. However, severe anaemia during hospitalisation is independently associated with an increased mortality over a 1 year period after discharge.

## Abbreviations

ADL: Activities of daily living
BMI: Body mass index
eGFR: Estimated glomerular filtration ratio
MMSE: Mini-Mental State Examination
